# Metabolites contributing to *Rhizoctonia solani* AG-1-IA maturation and sclerotial differentiation revealed by UPLC-QTOF-MS metabolomics

**DOI:** 10.1371/journal.pone.0177464

**Published:** 2017-05-10

**Authors:** Wenjin Hu, Xinli Pan, Hafiz Muhammad Khalid Abbas, Fengfeng Li, Wubei Dong

**Affiliations:** 1 Department of Plant Pathology, College of Plant Science and Technology and the Key Lab of Crop Disease Monitoring & Safety Control in Hubei Province, Huazhong Agricultural University, Wuhan, Hubei Province, China; 2 Department of Biochemical and Chemical Engineering, Technische Universität Dortmund, Dortmund, Germany; Fujian Agriculture and Forestry University, CHINA

## Abstract

*Rhizoctonia solani* is a causative agent of sheath blight, which results in huge economic losses every year. During its life cycle, the formation of sclerotia helps *Rhizoctonia solani* withstand a variety of unfavorable factors. Oxidative stress is a key factor that induces sclerotium formation. The differentiated and undifferentiated phenotypes of *R*. *solani* AG-1-IA were obtained by controlling aerial conditions. Metabolomics based on the mass spectrometry technique combined with multivariate and univariate analyses was used to investigate the metabolic variation in vegetative, differentiated and undifferentiated mycelia. Our results revealed that during maturation, the metabolic levels of N2-acetyl-L-ornithine, 3,1'-(OH)2-Gamma-carotene, (5Z,7E)-(1S,3R)-24,24-difluoro-24a-homo-9,10-seco-5,7,10(19)-cholestatrien-1,3,25-triol, stoloniferone O, PA(O-18:0/12:0), PA(P-16:0/14:0), PA(P-16:0/16:(19Z)) and PA(P-16:0/17:2(9Z,12Z)) were suppressed in both differentiated and undifferentiated mycelia. The concentrations of PE(20:1(11Z)/14:1(9Z)), PE(P-16:0/20:4(5Z,8Z,11Z,13E)(15OH[S])) and PS(12:0/18:1(9Z)) were increased in the differentiated group, while increased levels of N(gamma)-nitro-L-arginine, tenuazonic acid and 9S,10S,11R-trihydroxy-12Z,15Z-octadecadienoic acid were found in the undifferentiated group. Our results suggest that different levels of these metabolites may act as biomarkers for the developmental stages of *R*. *solani* AG-1-IA. Moreover, the mechanisms of sclerotium formation and mycelium differentiation were elucidated at the metabolic level.

## Introduction

*Rhizoctonia solani* is a notorious phytopathogenic basidiomycete fungus with a wide range of hosts and worldwide distribution. It causes massive economic losses of important crops, such as rice, maize and soybean, every year [1]. There are 14 anastomosis groups of *R*. *solani* that are further divided into inter-specific groups based on different host ranges, culture appearance or thiamine requirements [2]. Diseases caused by *R*. *solani* include sheath blight, banded leaf, aerial blight and brown patch [3]. The life cycle of *R*. *solani* includes the stages of vegetative growth and sclerotium formation. Therefore, *R*. *solani* is considered to be an asexual fungus, even though its conidia are occasionally observed. Sclerotium formation involves the formation of small and discrete initials, an increase in size with liquid droplets on the surface, surface delineation and internal consolidation, along with melanin deposition [4]. Sclerotia play an important role in the life cycle of *R*. *solani*, which enable it to survive under unfavorable environmental conditions, such as low temperature [5]. Sclerotium formation in *R*. *solani* is affected by environmental factors, such as nutrient supply, light, temperature, pH and aeration [6, 7]. Up-regulation of oxidative stress induces mycelia differentiation [8]. In 1997, it was reported for the first time that sclerotial differentiation in *Sclerotium rolfsii* is accompanied by an increase in peroxide level. A theory was proposed that fungi survive unfavorable conditions by transitioning from vegetative mycelia to mature differentiated sclerotia [9]. Substances that are capable of strengthening or weakening oxidative stress can promote or reduce sclerotia formation. Sclerotium production can be reduced when hydroxyl radical scavengers, such as *β-*carotene and ascorbic acid, are added to the medium [10–12]. Low concentrations of L-cysteine, L-homocysteine and glutathione inhibit the formation of sclerotia due to their ability to decrease the levels of oxidative stress [13–16]. Variations in the concentrations of oxidative stress-associated thiols are observed in sclerotiogenic phytopathogenic fungi, and a high thiol redox state is suggested to protect hyphae in the sclerotia [17]. The levels of the antioxidant enzymes superoxide dismutase (SOD) and xanthine oxidase (XO) are highly increased in differentiated sclerotiogenic fungi [18]. Hence, it can be concluded that oxidative stress is one of the key factors related to sclerotium formation in sclerotiogenic fungi. However, based on our limited knowledge, metabolic fluctuations involved in sclerotium formation have not yet been elucidated.

Several -omics tools have been used to study the process of *R*. *solani* mycelium maturation, which have proven to be efficient techniques. Proteomics studies revealed that during the maturation of sclerotia, 55 different types of proteins are differentially expressed and involved in various cellular functional metabolic pathways [19]. Genes and proteins associated with modifying host cell walls or host infection were revealed by transcriptomics and proteomics [20, 21]. In metabolomics studies, during the maturation of *R*. *solani* sclerotia, 116 metabolites were identified, and among them, the metabolic levels of α-α-trehalose, D-glucose, 9-(Z)-octadecenoic acids, 9,12-octadecadienoic acids, xylitol and glucitol were significantly changed [22]. *R*. *solani* sclerotia extract was shown to exhibit phytotoxic and antibacterial properties, and constituents isolated from the extract include phenolics, carboxylic acids, carbohydrates, fatty acids and amino acids [23]. Until now, metabolic investigations of the transition of *R*. *solani* AG-1-IA from vegetative growth to differentiated or undifferentiated maturation have not been reported.

In this research, we found that sclerotium formation was inhibited under conditions in which the plate was sealed with a layer of preservative film during the maturation process. For a comparison, sclerotium formation was normal in unsealed plates. This phenomenon indicates that sclerotium formation of *R*. *solani* AG-1-IA is induced under aerial conditions. Samples of *R*. *solani* AG-1-IA from the three groups (vegetative growth group (G1), the mature, undifferentiated group (G2) and the mature, differentiated group (G3)) were collected. Extracts of mycelia from these three groups were tested by ultra-performance liquid chromatography quadrupole time-of-flight mass spectrometry (UPLC-QTOF-MS) and analyzed using multivariate and univariate analyses. Characteristic metabolites that play key roles in the discrimination of these three groups were identified. Metabolic variations between vegetative and mature *R*. *solani* AG-1-IA, as well as variations between differentiated and undifferentiated *R*. *solani* AG-1-IA during maturation were investigated. This research provides metabolic information on the mechanism of sclerotium formation and may aid in the development of strategies for sclerotial fungus control.

## Materials and methods

### Maintenance of cultures and collection of samples

Cultures of *R*. *solani* isolate AG-1-IA were maintained on PDA (potato dextrose agar) at 4°C according to our previous research [24]. Agar plugs from actively growing margins of the colony were inoculated onto fresh PDA plates (14 cm in diameter and 30 ml PDA medium in each plate) and incubated at 28°C in the dark. Cultures were divided into 3 groups. In the first vegetative growing group (G1), cultures of vegetative mycelia from sealed plates were collected 36 hours post inoculation and ground into a fine powder using liquid nitrogen. The powder was then transferred to a 50 ml tube and stored at -80°C for further use. On the basis of aeration conditions, samples from the second and third groups were defined 48 hours post-inoculation when the front segment of the mycelia reached the edge of the Petri dish. The second undifferentiated group (G2) consisted of sealed plates with a layer of preservative film while the third differentiated group (G3) did not have a layer of preservative film. Sixty hours post-inoculation, samples were collected according to the same procedure used for the first group. Each group consisted of 5 replicates and each replicate consisted of mycelia from two plates.

### Preparation of samples

All samples were placed into a vacuum lyophilizer for two days and stored at -80°C. Fifty milligrams of each sample and 1.5 ml of methanol stock solution (HPLC purified, 0.1% v/v formic acid) were added to a 2 ml Eppendorf tube. After 2 min of vertexing, samples were extracted using a shaker (24°C at 120 rpm for 4 hours). After 20 min of sonication and the first centrifugation (4°C at 14,000 ×*g* for 20 min), 1 ml of supernatant was transferred into a new 2 ml Eppendorf tube and centrifuged for the second time. From each sample, 0.9 ml of supernatant from the second centrifugation was filtered with a 0.22 μm filter and transferred into auto-sampler vials. For UPLC-QTOF-MS analysis, each sample was injected once for each group.

### UPLC-QTOF-MS analysis

The Waters Ultra Performance Liquid Chromatography system used for metabolomics was equipped with an ACQUITY C_18_ column (10 cm × 2.1 mm, particle size 1.7 μm, Waters, USA). The column was eluted according to the following sequence with acetonitrile solution (A:B; A = water (0.1% formic acid), B = acetonitrile): 1 min, 99:1; 2 min, 80:20; 5 min, 60:40; 6 min, 45:55; 13 min, 20:80; 15 min, 5:95; 17 min, 5:95; 20 min, 99:1. The gradient duration was 20 min at a flow rate of 0.4 ml/min with the column temperature set at 45°C. One microliter of each sample was injected into the column. Mass spectrometry was performed on the Waters QTOF MS and operated in positive ion mode with a scan range from 50 to 1200 m/z. The desolvation gas flow was set to 500 l/h at a desolvation temperature of 400°C. The cone gas was set to 50 l/h and the source temperature was 100°C. The capillary voltage and cone voltage were set to 1000 and 30 V, respectively. The QTOF MS acquisition rate was set at 0.3 s with a 0 s inter-scan delay. Tune page was used to regulate the sample cone voltage. In the MS/MS experiments, argon was employed as the collision gas, and the collision energy was set to 6 eV. The MS collision low and high energy were set at 15.0 and 45.0 eV, respectively. Data were collected in centroid mode. All analyses were acquired using the lock spray, which was set at 20 s to ensure accuracy and reproducibility; leucine–enkephalin was used as the lock mass at a concentration of 0.8 ng/μL and a flow rate of 10 μL/min.

### Data processing, analysis and biomarker identification

The RAW-format data were processed using the DataBridge utility of the MassLynx software package (Waters). Four NetCDF files were generated from the original raw data, and the first two NetCDF files were used for statistical analysis and metabolite identification. The parameters of the XCMS process were as follows [25]: the peak picking method was the CentWave algorithm, the ppm was set at 15, the prefilter range was set between 2–200, the peak width was set between 5–12, the polarity was set as positive, the baseline was corrected using the Oribwarp method, and the method for grouping peaks was set as bw = 2, minfrac = 0.5 and mzwid = 0.015. Finally, the missing peaks were filled using default parameters. Spectra groups were calculated using the CAMERA [26] package with perfwhm = 0.6 and mzabs = 0.01. The threshold for the correlation coefficient was set to 0.75, and the parameters for finding additives were ran in positive ion mode.

After obtaining the peak list and intensity data, all signal intensities in each sample were normalized using the sum of all intensities. The intensity of each signal among samples was scaled using the Pareto method. To avoid bias errors introduced by dominating peaks, the unit variance scaling method was applied. Unit variance scaling was determined to be a good choice for both PCA and PLS-DA (S1 Fig). However, based on the reference of the related metabolomics research of other *R*. *solani* species [22], the Pareto scaling method was preferred. The R statistical computing environment was used for data analysis. PCA was first used for preliminary analysis, and the results are presented as score plots to observe the raw differences among groups. PLS-DA was then used for every two groups and the results are presented as score plots. For each PLS-DA model, the R2Y, Q2Y, root-mean-square error of estimation (RMSEE) value and 7-fold cross-validation were used to estimate model performance. The 7-fold cross-validation can guarantee the fair explanation of variations among 10 samples from two groups as well as reliability in the prediction of each PLS-DA model. During the process of each cross-validation test, samples were segmented into 7 groups by the "consecutive" method. Additionally, to determine whether each PLS-DA model was over-fitted, the permutation test (the number of permutation tests = 100) was applied. Variables with significant contributions to the separation of samples between groups were selected based on the variable influence on projection value (VIP > 3). These variables were then subjected to the univariate Welch’s t-test, and variables with significant changes were selected according to P < 0.01. The corresponding fold change values were also calculated. The parent ions were picked from the results derived from the multivariate and univariate analyses by setting the parent ions as [M+H] + or [M+Na] +. Taking into account the lack of structural information of metabolites in *R*. *solani*, the structure determination of metabolites was mostly dependent on the online database METLIN [27] and lipid maps [28]. The m/z and intensity values of each parent ion and its associated fragment ions were derived from the annotation of the CAMERA package. The m/z value of each parent ion was used to retrieve the matched metabolites in the online database, where the relative mass deviation in the database search was 30 and the searching mode was set as [M+H] + or [M+Na] +. The SMILES structure of each candidate metabolite was retrieved using the PubChem compound accession identifier (CID) corresponding to its name [29]. Each SMILES structure was fragmented in silico using the MetFrag package (2.0) [30]. Metabolites with at least 3 fragments, the m/z values of which matched the m/z values of the current parent ion and its associated fragment ions, were retained. The remaining metabolites were scored based on the m/z value, as well as the signal intensity of the matched peaks using MetFrag when the absolute mass deviation in the fragment peak match was limited to 0.01 and the relative mass deviation in the fragment peak match was 5.0. Finally, the metabolite with the highest score was selected as the right solution. We believe that such a process can make the confirmation more convincing.

## Results

### Differences between the three phenotypes of mycelia

**Fig 1:**
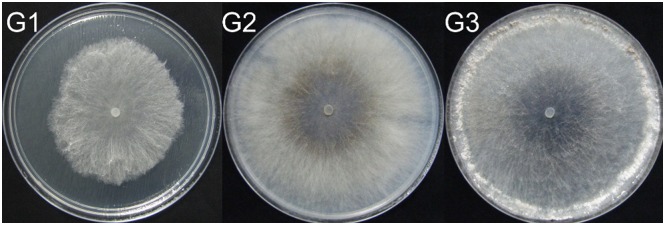
Phenotypes of vegetative (G1), undifferentiated (G2) and differentiated (G3) *Rhizoctonia solani* AG-1-IA in potato dextrose agar plates. G1: Culturing 36 hours post-inoculation while sealing the plate with a layer of preservative film. G2: Culturing 60 hours post-inoculation while sealing the plate with a layer of preservative film continuously. G3: Culturing 60 hours post-inoculation without sealing the plate beginning from 48 hours to 60 hours (the preservative film was removed at 48 hours). Differentiated sclerotia were formed in G3 due to unlimited aeration. After 48 hours of growth, the mycelia reached the edge of the Petri dish, but at 60 hours, the mycelia remained undifferentiated in G2 due to isolated aeration.

The three representative phenotypes of mycelia are shown in Fig 1. The sample from the first group (G1) was incubated for 36 hours. In the G1 Petri dish, the front segment of mycelia did not reach the margins of the Petri dish, but the white aerial mycelia grew vigorously. Samples from the second group (G2) and third group (G3) were cultured and harvested at the same time after 60 hours of incubation. Mycelia of the G2 group were less vigorous than the vegetative growing mycelia in G1, and dark brown pigment was produced in the middle of the culture dish. No sclerotium was observed in G2. In G3, mycelia were aggregated at the margins of the Petri dish to produce sclerotia, while the middle of the Petri dish became transparent. Differences between the undifferentiated G2 and differentiated G3 mycelia were mainly attributed to the aeration conditions in the respective Petri dishes because the sealing layer of preservative film in the differentiated group G3 was removed while the dishes in the undifferentiated group G2 remained sealed with a layer of preservative film after the front segment of mycelia reached the margin of the plates. To explore the mechanism that caused these phenotypic differences, the metabolite levels in these three groups were investigated.

### Statistical variations in the three types of mycelia

**Fig 2:**
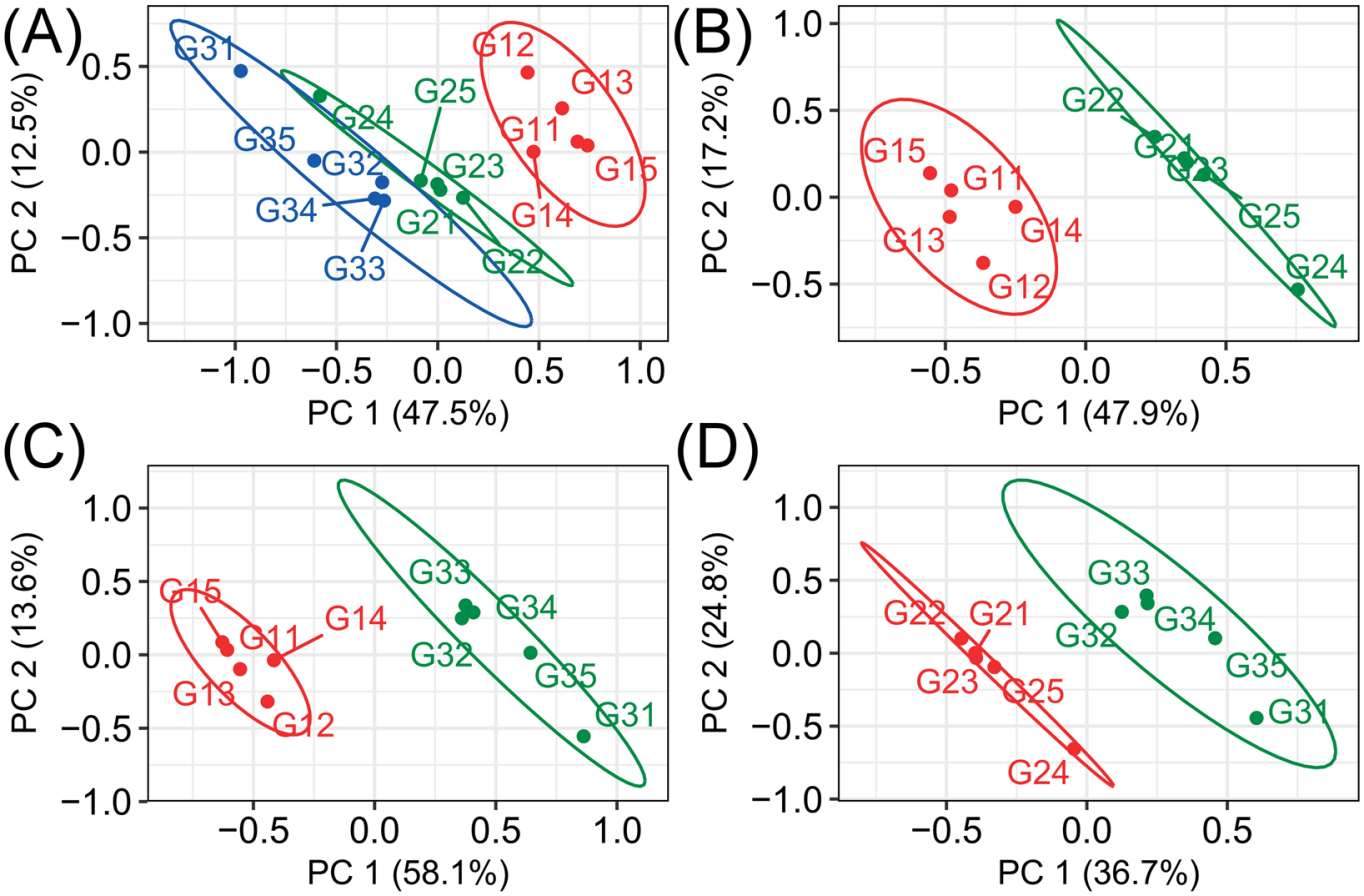
Score plots derived from principal component analysis (PCA) and partial least squares discriminant analysis (PLS-DA). (A): Score plots based on the first two components (PC1 vs PC2) derived from the PCA results. (B), (C) and (D): Score plots based on the first two latent components derived from the corresponding PLS-DA model for the three comparisons (G2 vs G1, G3 vs G1 and G3 vs G2). According to the paired comparison, the separation between every two groups was clear. The R2X, R2Y, Q2Y and RMSEE values in the PLS-DA models for groups G2 and G1 were 65.1%, 97.6%, 93.9% and 0.022, respectively. The R2X, R2Y, Q2Y and RMSEE values in the PLS-DA models for groups G3 and G1 were 71.7%, 98.9%, 97.1% and 0.012, respectively. The R2X, R2Y, Q2Y and RMSEE values in the PLS-DA models for groups G3 and G2 were 61.5%, 98.1%, 87.8% and 0.02, respectively. The ellipse for each group represented Hotelling’s T^2^ 95% confidence interval.

The *R*. *solani* AG-1-IA mycelia of each group were extracted and analyzed by UPLC-QTOF-MS, and the original peaks were picked and annotated by XCMS and CAMERA packages (S1 Table). The data were first normalized in each row (sample) and scaled in each column (variable). Next, unsupervised principal component analysis (PCA) was used to analyze the entire dataset. The PCA score plots are shown in Fig 2(A). The results showed that 60% of the variance among all variables could be explained by the first two principal components, where the first principal component accounted for 47.5% of the total and the second accounted for 12.5% of the remaining components. These score plots demonstrated that the three groups could be clearly separated based on the first two principal components, even though the G2 and G3 groups were close to each other, revealing a clear difference between the vegetative mycelia and the two mature mycelia. Differences were also observed between the differentiated G3 and undifferentiated G2 groups. Nevertheless, the primary results obtained by PCA remained obscure. Supervised PLS-DA was used to analyze each pair of the three groups. Variables that made important contributions to the differences between the three phenotypic groups (VIP > 3) were selected and their significances were validated using the univariate Welch’s t-test analysis (P-value < 0.01). Based on the statistical results and the annotated mass features, the parent ion masses were selected and used to search for metabolites. Multiple metabolites corresponding to each parent ion were scored using the MetFrag package (S2 Table). The right solution for each parent ion mass is listed in Table 1. A total of 35 metabolites were associated with three different phenotypes: six were identified as oleic or linolenic acids and ten were identified as glycerophospholipids. N2-acetyl-L-ornithine and N(gamma)-nitro-L-arginine were determined to be involved in the metabolism of ornithine and putrescine, respectively. 3,1'-(OH)2-Gamma-carotene and 5,6-dihydro-5,6-dihydroxy-y,y-carotene were determined to belong to carotenoids; stoloniferone O was determined to belong to ergosterols; (5Z,7E)-(1S,3R)-24,24-difluoro-24a-homo-9,10-seco-5,7,10(19)-cholestatrien-1,3,25-triol was determined to belong to vitamins; and tenuazonic acid was determined to belong to phytotoxin.

**Table 1:** Identification of the metabolites.

Ion mass	Adduct	Mass	Compound name
165.0410	[M+H]+	164.034	2-dehydro-D-xylonate
175.1053	[M+H]+	174.099	N2-acetyl-L-ornithine
186.0923	[M+H]+	185.085	(S)-N-(4,5-dihydro-1-methyl-4-oxo-1H-imidazol-2-yl)alanine
198.1102	[M+H]+	197.103	tenuazonic acid
220.1049	[M+H]+	219.100	N(gamma)-nitro-L-arginine
295.2158	[M+H]+	294.206	IDFP
313.2271	[M+H]+	312.222	fruticosonine
333.1939	[M+Na]+	310.203	9-hydroperoxy-10E,12,15Z-octadecatrienoic acid
349.1899	[M+Na]+	326.201	2R-hydroperoxy-9Z,12Z,15Z-octadecatrienoic acid
351.2059	[M+Na]+	328.215	9S,10S,11R-trihydroxy-12Z,15Z-octadecadienoic acid
353.2212	[M+Na]+	330.234	9S,10S,11R-trihydroxy-12Z-octadecenoic acid
365.0973	[M+Na]+	342.106	6,8-di-C-methylkaempferol 3,7-dimethyl ether
365.2209	[M+H]+	364.214	6 alpha-fluoro-11beta,17-dihydroxypregn-4-ene-3,20-dione
367.2371	[M+Na]+	344.243	12,13-dihydroxy-11-methoxy-9-octadecenoic acid
369.2164	[M+Na]+	346.223	9,10-dihydroxy-13-hydroperoxy-11-octadecenoic acid
427.3145	[M+H]+	426.308	stoloniferone O
467.3315	[M+H]+	466.324	(5Z,7E,22E,24E)-(1S,3R)-24a-homo-9,10-seco-5,7,10(19),22,24-cholestapentaene-1,3,25-triol
505.1745	[M+Na]+	482.186	melleolide D
522.2983	[M+H]+	521.290	PS(18:2(9Z,12Z)/0:0)
571.4366	[M+H]+	570.430	3,1'-(OH)2-Gamma-carotene
573.4523	[M+H]+	572.446	5,6-dihydro-5,6-dihydroxy-y,y-carotene
607.4591	[M+H]+	606.451	PA(O-18:0/12:0)
612.4347	[M+Na]+	589.445	CerP(d18:1/14:0)
627.4259	[M+Na]+	604.439	PA(P-16:0/14:0)
629.4410	[M+Na]+	606.453	staphidine
631.4563	[M+H]+	630.447	PA(P-16:0/16:1(9Z))
647.4534	[M+Na]+	624.462	DG(15:1(9Z)/22:6(4Z,7Z,10Z,13Z,16Z,19Z)/0:0)[iso2]
661.4687	[M+Na]+	638.481	DG(16:1(9Z)/22:6(4Z,7Z,10Z,13Z,16Z,19Z)/0:0)[iso2]
665.4642	[M+Na]+	642.473	PA(P-16:0/17:2(9Z,12Z))
706.4618	[M+H]+	705.452	PS(12:0/18:1(9Z))
716.5305	[M+H]+	715.523	PE(20:1(11Z)/14:1(9Z))
736.6176	[M+H]+	735.608	DGTS(16:0/18:2(9Z,12Z))
740.5315	[M+H]+	739.525	PE(P-16:0/20:4(5Z,8Z,11Z,13E)(15OH[S]))
758.6003	[M+H]+	757.585	PC(O-18:0/17:2(9Z,12Z))
818.5483	[M+H]+	817.538	PC(17:1(9Z)/22:6(4Z,7Z,10Z,13Z,16Z,19Z))

Comparisons among every two groups were carried out using PLS-DA. The predictive components for each PLS-DA model were selected automatically, and the corresponding loading plots for three PLS-DA models were constructed (S2 Fig). The PLS-DA models for groups G1 and G2 were built based on three components, and the score plot based on the first two components is presented in Fig 2(B), which shows that groups G1 and G2 are clearly separated. No outlier was found based on a 95% confidence interval for each group. Of the total variance of variable X, 65.1% was explained by the first two latent principal components, where the first principal component accounted for 47.9% of the total variance and the second accounted for 17.2% of the remaining variance. Additionally, 97.6% of the total variance of variable Y (R2Y) was explained by the first two components, and the predicative ability (Q2Y) by the first two components in this model was calculated as 93.9%, suggesting that this model generated good predictions. The PLS-DA models for groups G1 and G3 were built on the first two components and the score plot is presented in Fig 2(C), which shows a clear separation between the two groups, where the first two components explained 71.7% of the total variance; the first accounted for 58.1% of the total variance and the second accounted for 13.6% of the total variance. A total of 98.9% of the variance in variable Y (R2Y) was also explained by the first two components and the ability of prediction in this model (Q2Y) was calculated as 97.1%. The PLS-DA models for groups G2 and G3 were built on the first two components and the score plot is presented in Fig 2(D). The PLS model demonstrated reliable ability in fitness and prediction because the first two components derived from it described 61.5% of the variation in the response of X and 98.1% of the variation in the response of Y (R2Y), and predicted 87.8% of the variation in the response of Y (Q2Y). Furthermore, the RMSEE values in the PLS-DA models for G1 vs G2, G1 vs G3 and G2 vs G3 were 0.022, 0.012 and 0.02, respectively, and the permutation tests proved that each PLS-DA model was not over-fitted (S3 Fig). These results suggest that the PLS-DA models are qualified and reliable. Significantly altered metabolites from the combined statistical analyses were selected and fold change values for each pair comparison of the three groups were calculated. The metabolites that were significantly altered among the three groups are presented in Table 2.

**Table 2:** Concentration ratio of the same metabolite compared among mycelium groups.

Name	G31	G21	G32
2-dehydro-D-xylonate	0.4226 **	0.5068 **	0.8337
N2-acetyl-L-ornithine	0.0570 **	0.3640 **	0.1566 **
(S)-N-(4,5-dihydro-1-methyl-4-oxo-1H-imidazol-2-yl)alanine	3.6692 **	2.6615 *	1.3786
tenuazonic acid	0.1657 **	1.8681 **	0.0887 **
N(gamma)-Nitro-L-arginine	0.2498 **	1.5559 *	0.1606 **
IDFP	0.2911 **	0.4834 **	0.6022
fruticosonine	0.7384	1.3500 *	0.5469 **
9-hydroperoxy-10E,12,15Z-octadecatrienoic acid	0.0789 **	0.1081 **	0.7299
2R-hydroperoxy-9Z,12Z,15Z-octadecatrienoic acid	0.3591 **	0.7896	0.4547
9S,10S,11R-trihydroxy-12Z,15Z-octadecadienoic acid	0.5665 *	1.9188 **	0.2952 **
9S,10S,11R-trihydroxy-12Z-octadecenoic acid	0.2501 **	0.4054 **	0.6169
6,8-di-C-methylkaempferol 3,7-dimethyl ether	1.8646 *	2.9026 **	0.6424 *
6 alpha-fluoro-11beta,17-dihydroxypregn-4-ene-3,20-dione	0.1815 **	0.1593 **	1.1395
12,13-dihydroxy-11-methoxy-9-octadecenoic acid	0.5326 *	0.5199 **	1.0245
9,10-dihydroxy-13-hydroperoxy-11-octadecenoic acid	0.1133 **	0.6911	0.1640
stoloniferone O	0.5218 **	0.7291 **	0.7156 **
(5Z,7E,22E,24E)-(1S,3R)-24a-homo-9,10-seco-5,7,10(19),22,24-cholestapentaene-1,3,25-triol	0.2344 **	0.5986 *	0.3915 *
melleolide D	3.1957 *	2.5503 **	1.2531
PS(18:2(9Z,12Z)/0:0)	0.3240 **	0.3267 **	0.9917
3,1'-(OH)2-Gamma-carotene	0.0897 **	0.1514 **	0.5922
5,6-dihydro-5,6-dihydroxy-y,y-carotene	0.3819 **	0.7045	0.5421
PA(O-18:0/12:0)	0.1391 **	0.2485 **	0.5597
CerP(d18:1/14:0)	0.1098 **	0.1754 **	0.6261
PA(P-16:0/14:0)	0.0451 **	0.1349 *	0.3343 *
staphidine	0.1012 **	0.3293 **	0.3074 *
PA(P-16:0/16:(19Z))	0.1221 **	0.3850 **	0.3171 *
DG(15:1(9Z)/22:6(4Z,7Z,10Z,13Z,16Z,19Z)/0:0)[iso2]	0.1396 **	0.3911 *	0.3570 *
DG(16:1(9Z)/22:64Z,7Z,10Z,13Z,16Z,19Z)/0:0)[iso2]	0.4360 **	0.8919	0.4888
PA(P-16:0/17:2(9Z,12Z))	0.0148 **	0.0455 **	0.3246
PS(12:0/18:1(9Z))	2.3171 **	1.6306 **	1.4210 *
PE(20:1(11Z)/14:1(9Z))	14.1878 **	10.2179	1.3885
DGTS(16:0/18:2(9Z,12Z))	3.9315 **	3.4976 *	1.1240
PE(P-16:0/20:4(5Z,8Z,11Z,13E)(15OH[S]))	10.8887 **	7.1047	1.5326
PC(O-18:0/17:2(9Z,12Z))	3.0710 *	3.7417 **	0.8207
PC(17:1(9Z)/22:6(4Z,7Z,10Z,13Z,16Z,19Z))	0.4875 **	0.9872	0.4938

G21: Each value represents the ratio of the average concentration of a given metabolite in group 2 divided by the average concentration of the same metabolite in group 1. G31 and G32: Each value represents the ratio of the average concentration of a given metabolite in group 3 divided by the average concentration of the same metabolite in groups 1 and 2, respectively.

**: p-value < 0.01.

*: p-value < 0.05.

### Use of metabolites associated with phenotypic differences as biomarkers

Compared to the vegetative mycelia group G1, the metabolites with significantly (p-value < 0.01) decreased concentrations in both mature groups (G2 and G3) were: 2-dehydro-D-xylonate, N2-acetyl-L-ornithine, IDFP, 9-hydroperoxy-10E,12,15Z-octadecatrienoic acid, 9S,10S,11R-trihydroxy-12Z-octadecenoic acid, 6 alpha-fluoro-11beta,17-dihydroxypregn-4-ene-3,20-dione, stoloniferone O, PS(18:2(9Z,12Z)/0:0), 3,1’-(OH)2-Gamma-carotene, PA(O-18:0/12:0), CerP(d18:1/14:0), staphidine, PA(P-16:0/16:(19Z)) and PA(P-16:0/17:2(9Z,12Z)). Additionally, the concentrations of metabolites (5Z,7E,22E,24E)-(1S,3R)-24a-homo-9,10-seco-5,7,10(19),22,24-cholestapentaene-1,3,25-triol, PA(P-16:0/14:0) and DG(15:1(9Z)/22:6(4Z,7Z,10Z,13Z,16Z,19Z)/0:0)[iso2] were significantly decreased in the differentiated group G3 (p-value < 0.01) and the undifferentiated group G2 (p-value < 0.05). Among them, when comparing the two mature groups, metabolites with higher (p-value < 0.05) concentrations in the undifferentiated group G2 were: N2-acetyl-L-ornithine, stoloniferone O, (5Z,7E,22E,24E)-(1S,3R)-24a-homo-9,10-seco-5,7,10(19),22,24-cholestapentaene-1,3,25-triol, staphidine, PA(P-16:0/16:(19Z)) and DG(15:1(9Z)/22:6(4Z,7Z,10Z,13Z,16Z,19Z)/0:0)[iso2]. Compared to the vegetative mycelia group, the concentrations of 2R-hydroperoxy-9Z,12Z,15Z-octadecatrienoic acid, 9,10-dihydroxy-13-hydroperoxy-11-octadecenoic acid, 5,6-dihydro-5,6-dihydroxy-y,y-carotene, DG(16:1(9Z)/22:64Z,7Z,10Z,13Z,16Z,19Z)/0:0)[iso2] and PC(17:1(9Z)/22:6(4Z,7Z,10Z,13Z,16Z,19Z)) were decreased only in the differentiated group.

**Fig 3:**
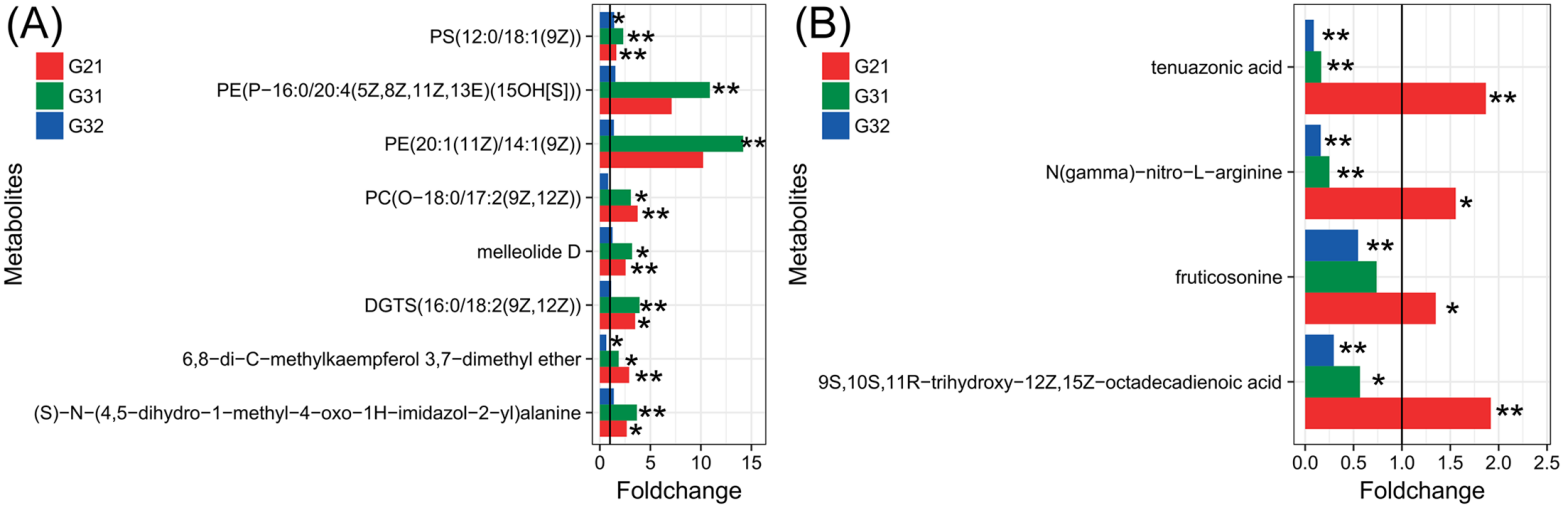
Bar plots of the significantly altered concentration ratio of the same metabolite compared among mycelium groups. (A): Metabolites with increased concentrations in both mature groups. (B): Metabolites with increased concentrations in the undifferentiated group but decreased concentrations in the differentiated group. G21: Each value represents the ratio of the average concentration of a given metabolite in group 2 divided by the average concentration of the same metabolite in group 1. G31 and G32: Each value represents the ratio of the average concentration of a given metabolite in group 3 divided by the average concentration of the same metabolite in groups 1 and 2, respectively. **: p-value < 0.01. *: p-value < 0.05. The black vertical line indicates a fold change equal to 1.

The metabolites with increased concentrations in both mature groups are presented in Fig 3(A). The concentrations of metabolites including (S)-N-(4,5-dihydro-1-methyl-4-oxo-1H-imidazol-2-yl) alanine (p-value < 0.05 when G2 vs G1), 6,8-di-C-methylkaempferol 3,7-dimethyl ether (p-value < 0.05 when G3 vs G1), melleolide D (p-value < 0.05 when G3 vs G1), PS(12:0/18:1(9Z)), DGTS(16:0/18:2(9Z,12Z)) (p-value < 0.05 when G2 vs G1) and PC(O-18:0/17:2(9Z,12Z)) (p-value < 0.05 when G3 vs G1) were higher in the two mature groups. Among them, the concentration of 6,8-di-C-methylkaempferol 3,7-dimethyl ether remained (p-value < 0.05) increased in the undifferentiated group G2, while the concentration of PS(12:0/18:1(9Z)) was higher in the differentiated group G3 (p-value < 0.05). Additionally, the concentrations of PE(20:1(11Z)/14:1(9Z)) and PE(P-16:0/20:4(5Z,8Z,11Z,13E)(15OH[S])) were only significantly higher in the differentiated group G3 compared to the vegetative mycelia group.

The two mature groups were compared to the vegetative groups and the variations in concentrations of metabolites are presented in Fig 3(B). The concentrations of tenuazonic acid, N(gamma)-nitro-L-arginine (p-value < 0.05), 9S,10S,11R-trihydroxy-12Z,15Z-octadecadienoic acid and fruticosonine (p-value < 0.05) were increased in the undifferentiated group G2, while all of them except fruticosonine were decreased in the differentiated group G3. Thus, we believe that the levels of these metabolites could be used to differentiate the developmental stages of vegetative or mature mycelia, suggesting that changes in the concentration of these metabolites could be used as a biomarker to determine whether the sclerotium has formed.

## Discussion

Three mycelia groups that represent three different types of mycelia collected at two different time points were examined in this study. The first group (G1) was collected 36 hours after inoculation, and the second and third groups were collected 60 hours after inoculation. When mycelia growth reached the margins of the Petri dishes 48 hours after inoculation, the culture in the second group (G2) remained sealed with a layer of preservative film around the plate edge to avoid aeration while, in the third group (G3), the sealing preservative film was removed to provide unlimited aeration. The three groups were phenotypically different. The marginal mycelial growth of the vegetative group G1 was white and vigorous. The mycelia of the undifferentiated group G2 was sparse and a dark brown pigment was secreted in the middle of the mycelial colony where no sclerotium formation was observed. In the differentiated group G3, the mycelia aggregated into white clusters at the edge of the culture plate to form a sclerotium and a hyphae colony appeared transparent in the middle of the plate. Incubation time was the major factor contributing to the differences between vegetative growth and mature mycelial phenotypes, whereas aeration was the main factor that caused differences in the two mature mycelial phenotypes. Previous studies have shown that oxygen in the air plays an important role in the formation of sclerotia [8]. To examine potential metabolic variations in these three phenotypes and to link the metabolism in each group with the corresponding phenotype, we collected samples of the three mycelial phenotypes and used UPLC-QTOF-MS combined with multivariate and univariate analyses to classify them at the metabolic level.

The concentration of N2-acetyl-L-ornithine was significantly reduced in both types of mature mycelia, regardless of whether sclerotia had formed. Additionally, the content of N2-acetyl-L-ornithine in undifferentiated mycelia was significantly higher than that in differentiated mycelia. N2-acetyl-L-ornithine is a product of glutamate metabolism and can be used to synthesize ornithine. The decarboxylation of ornithine catalyzed by the decarboxylase leads to the production of putrescine. The activity and concentration of ornithine decarboxylase are decreased when fungal growth terminates due to a depletion of glucose, and a sharp decrease in ornithine decarboxylase activity and putrescine content can be detected during the transition from vegetative mycelia to mature sclerotia in *Sclerotium rolfii* [31]. Our results prove that the synthesis of ornithine is decreased due to the reduced concentration of N2-acetyl-L-ornithine in both types of mature mycelia. Furthermore, different concentrations of N2-acetyl-L-ornithine were observed, with the highest level observed in the vegetative growing mycelia, less in the sparse undifferentiated mycelia and the least in the differentiated mycelia. These observations suggest that vigorous growth of *R*. *solani* AG-1-IA mycelia requires a high level of N2-acetyl-L-ornithine, while sclerotia formation requires the inhibition of N2-acetyl-L-ornithine metabolism.

The concentration of 3,1'-(OH)2-Gamma-carotene was significantly reduced in both groups of mature mycelia. However, there was no significant difference in the concentration of 3,1'-(OH)2-Gamma-carotene between the differentiated and undifferentiated phenotypes. Moreover, the concentration of 5,6-dihydro-5,6-dihydroxy-y,y-carotene was significantly decreased only in the differentiated group. Gamma-carotene can be converted into beta-carotene. As a hydroxyl radical scavenger, beta-carotene can inhibit sclerotium differentiation [10]. Our results suggest that the metabolism of 3,1'-(OH)2-Gamma-carotene is related only to the maturation of mycelia but not to sclerotia formation. However, the metabolism of 5,6-dihydro-5,6-dihydroxy-y,y-carotene may play an important role in sclerotium formation. The concentration of (5Z,7E)-(1S,3R)-24,24-difluoro-24a-homo-9,10-seco-5,7,10(19)-cholestatrien-1,3,25-triol, which belongs to the vitamin family, was reduced in the differentiated and undifferentiated groups compared to the vegetative group. Vitamin B6 can quench reactive oxygen species (ROS) when the fungus is exposed to environmental stress [32]. Genes associated with vitamin B6 synthesis are significantly up-regulated under oxidative stress in *R*. *solani* [33]. Based on these results, especially the contribution of ROS to sclerotium formation, we speculate that vitamin metabolism plays an important role in sclerotium formation.

Compared to the vegetative growing mycelia, the concentration of N(gamma)-nitro-L-arginine was reduced in the differentiated group, while its concentration was increased in the undifferentiated group. N(gamma)-Nitro-L-arginine is an inhibitor of nitric oxide (NO) synthetase and can be used to prevent glutamate toxicity [34]. NO is able to regulate morphogenesis and the secondary metabolism in *Aspergillus nidulans* [35]. A decrease in the concentration of N(gamma)-nitro-L-arginine suggests that the production of NO was promoted since NO synthetase activity was not inhibited when differentiation began in *R*. *solani* AG-1-IA. In the case of undifferentiated mycelia, the increased concentration of N(gamma)-nitro-L-arginine suggests that the metabolism of NO was suppressed. These results indicate that NO metabolism plays a key role in sclerotium formation. Similar metabolic behavior was also found regarding tenuazonic acid. Tenuazonic acid was first isolated from the plant pathogenic fungus *Alternaria tenuis* [36], which is also produced in the plant pathogens *Phoma sorghina* and *Magnaporthe oryzae* [37, 38]. Tenuazonic acid inhibits protein synthesis by suppressing the release of new proteins [39] and has been reported to exhibit antitumor, antitubercular, antibacterial, antiviral and phytotoxic activities [40–43]. This is the first report that tenuazonic acid also occurs in *R*. *solani* AG-1-IA. The concentration of tenuazonic acid was significantly higher in the undifferentiated group than in the vegetative and differentiated groups, suggesting that the concentration of tenuazonic acid is lower when hyphae grow normally and during the process of sclerotium formation. When sclerotium formation was interrupted by isolated aeration, the concentration of tenuazonic acid was significantly increased in the mature mycelia. However, until now, the role of tenuazonic acid in sclerotia formation remained unclear. The metabolic pathways involved in tenuazonic acid also remain unknown; however, it has been established that the production of tenuazonic acid is correlated with the expression of a fungal NRPS–PKS hybrid enzyme [44].

The concentration of linoleic acid varied in the two mature groups. The concentrations of 9-hydroperoxy-10E,12,15Z-octadecatrienoic acid, 9S,10S,11R-trihydroxy-12Z-octadecenoic acid and 12,13-dihydroxy-11-methoxy-9-octadecenoic acid were lower in both mature groups than in the vegetative growing group. Moreover, the concentrations of 2R-hydroperoxy-9Z,12Z,15Z-octadecatrienoic acid and 9,10-dihydroxy-13-hydroperoxy-11-octadecenoic acid were significantly lower only in the differentiated group compared to the vegetative growing group. In particular, the concentration of 9S,10S,11R-trihydroxy-12Z,15Z-octadecadienoic acid was significantly higher in the undifferentiated mycelia and lower in the differentiated mycelia. Approximately 94–98% of the fatty acids in the whole cell consist mostly of palmitic, oleic and linoleic acids [45]. Spore development can be induced in *Aspergillus spp* by hydroperoxylinoleic acids [46]. Our results suggest that sclerotium formation in *R*. *solani* AG-1-IA is accompanied by changes in oleic or linoleic acid content. Furthermore, the different concentrations of 9S,10S,11R-trihydroxy-12Z,15Z-octadecadienoic acid in the undifferentiated and differentiated groups suggest that the involved pathways affect sclerotium formation.

The expression of stoloniferone O was decreased in both mature groups. Stoloniferone O belongs to a family of ergosterols and is isolated from marine microorganisms [47]. During sclerotia formation, the expression of ergosterol-related proteins is significantly changed in *Sclerotinia sclerotiorum* [48]. Ergosterol is a component of fungal cell membranes [49, 50]. Metabolites in the sterol metabolic pathway are believed to regulate lipid metabolism [51]. Our results prove this hypothesis, since altered concentrations of glycerophospholipids were detected in both mature groups. When comparing the vegetative and mature groups, the concentrations of PS(12:0/18:1(9Z)) and PC(O-18:0/17:2(9Z,12Z)) were up-regulated in both mature groups. However, the concentration of PS(12:0/18:1(9Z)) was higher in the differentiated group than in the undifferentiated group. Additionally, the concentrations of PE(P-16:0/20:4(5Z,8Z,11Z,13E)(15OH[S])) and PE(20:1(11Z)/14:1(9Z)) were significantly higher only in the differentiated group. The levels of PA(O-18:0/12:0), PA(P-16:0/14:0), PA(P-16:0/16:(19Z)) and PA(P-16:0/17:2(9Z,12Z)) were lower in both mature groups. Glycerophospholipids can have many combinations of fatty acids with diverse lengths and saturation status. In glycerophospholipids, occupation of the glycerol substitution sites by phosphate moieties, phosphorylethanolamine moieties, phosphorylserine moieties or phosphorylcholine moieties leads to the production of glycerophosphates (e.g., PA), glycerophosphoethanolamines (e.g., PE), glycerophosphoserines (e.g., PS) or glycerophosphocholines (e.g., PC), respectively. Furthermore, the synthesis of PE, PS or PC requires diethanolamine, serine or choline, respectively, as substrates. PA can be transformed to PS or PE. PE synthesis can occur via the decarboxylation of PS. PS biosynthesis involves an exchange reaction of serine for ethanolamine in PE. PC can be synthesized via the conversion of either PS or PE. A decrease in the concentration of PA agrees with the concentration changes in oleic or linoleic acid discussed above, since PA synthesis requires oleic acid, linoleic acid or palmitic acid as a substrate. Increases in the concentrations of PE(20:1(11Z)/14:1(9Z)), PE(P-16:0/20:4(5Z,8Z,11Z,13E)(15OH[S])), and PS(12:0/18:1(9Z)) in the differentiated group suggests that the associated metabolic pathways are required for sclerotium formation.

## Conclusion

In our research, differentiation of mature *R*. *solani* AG-1-IA was affected by aerial conditions. Obvious phenotypic differences were observed among vegetative, undifferentiated and differentiated mycelia. Metabolomics analysis based on UPLC-QTOF-MS combined with multivariate and univariate statistics was proven to be an efficient tool for revealing metabolic differences among these three types of mycelia. Our results revealed that when *R*. *solani* AG-1-IA mycelia transitioned from vegetative growth to maturation, the metabolic levels of N2-acetyl-L-ornithine, 3,1'-(OH)2-Gamma-carotene, (5Z,7E)-(1S,3R)-24,24-difluoro-24a-homo-9,10-seco-5,7,10(19)-cholestatrien-1,3,25-triol, stoloniferone O, PA(O-18:0/12:0), PA(P-16:0/14:0), PA(P-16:0/16:(19Z)) and PA(P-16:0/17:2(9Z,12Z)) were decreased. When *R*. *solani* AG-1-IA mature mycelia began differentiation, the metabolic levels of PE(20:1(11Z)/14:1(9Z)), PE(P-16:0/20:4(5Z,8Z,11Z,13E)(15OH[S])) and PS(12:0/18:1(9Z)) were increased. When mature mycelia did not form the sclerotia, the metabolic levels of N(gamma)-nitro-L-arginine, tenuazonic acid and 9S,10S,11R-trihydroxy-12Z,15Z-octadecadienoic acid were increased. An increase in the levels of N(gamma)-nitro-L-arginine, tenuazonic acid and 9S,10S,11R-trihydroxy-12Z,15Z-octadecadienoic acid can be used as an indicator for mature *R*. *solani* AG-1-IA in the undifferentiated state, whereas an increase in the levels of PE(20:1(11Z)/14:1(9Z)), PE(P-16:0/20:4(5Z,8Z,11Z,13E)(15OH[S])) and PS(12:0/18:1(9Z)) can be used as an indicator for mature *R*. *solani* AG-1-IA in the differentiated state. Interestingly, tenuazonic acid, as a toxin, was detected in *R*. *solani* AG-1-IA mycelia extract for the first time.

## Supporting information

S1 Fig Score plots of principal component analysis (PCA) and partial least square discriminant analysis (PLS-DA) for the data scaled by the unit variance method. (A): Score plots based on the first two components (PC1 vs PC2) derived from the PCA result. (B), (C), and (D): Score plots based on the first two latent components derived from the corresponding PLS-DA model for the three comparisons (G2 vs G1, G3 vs G1 and G3 vs G2). The paired comparison suggested that the separation between every two groups was clear. The R2X, R2Y, Q2Y and RMSEE in PLS-DA models for groups G2 and G1 were 58.2%, 98.9%, 94.9% and 0.013, respectively. The R2X, R2Y, Q2Y and RMSEE in PLS-DA models for groups G3 and G1 were 62.6%, 99.6%, 98% and 0.039, respectively. The R2X, R2Y, Q2Y and RMSEE in PLS-DA models for groups G3 and G2 were 57.4%, 99.6%, 94.5% and 0.037, respectively. The ellipse for each group represented the Hotelling’s T^2^ 95% confidence interval.
(TIF)

S2 Fig Loading plots for three PLS-DA models. (A), (B), and (C): Loading plots based on the first two latent components derived from the corresponding PLS-DA model for the three comparisons (G2 vs G1, G3 vs G1 and G3 vs G2). The points which exhibited strong contribution to the construction of each PLS-DA model were labeled with the VIP values (VIP > 3).
(TIF)

S3 Fig Plots for permutation tests in PLS-DA models. (A), (B) and (C) were presented based on the permutation tests for PLS-DA models (G2 vs G1, G3 vs G1 and G3 vs G2), respectively. The R2Y and Q2Y values in each model were proved to be significant, which suggested that each PLS-DA model was not over-fitted.
(TIF)

S1 Table Raw dataset of the UPLC-QTOF-MS test. The dataset was outputted after that the original data were treated by the XCMS and CAMERA packages in R environment.
(CSV)

S2 Table Scores of candidates corresponding to each parent ion mass. Candidate for each parent ion mass was scored using the MetFrag package in R environment.
(CSV)
