# Supplementary figures and images for: Metabolites contributing to Rhizoctonia solani AG-1-IA maturation and sclerotial differentiation revealed by UPLC-QTOF-MS metabolomics
Source: PLoS One. 2017 May 10;12(5):e0177464. doi: 10.1371/journal.pone.0177464 (PMC5425210; doi:10.1371/journal.pone.0177464)

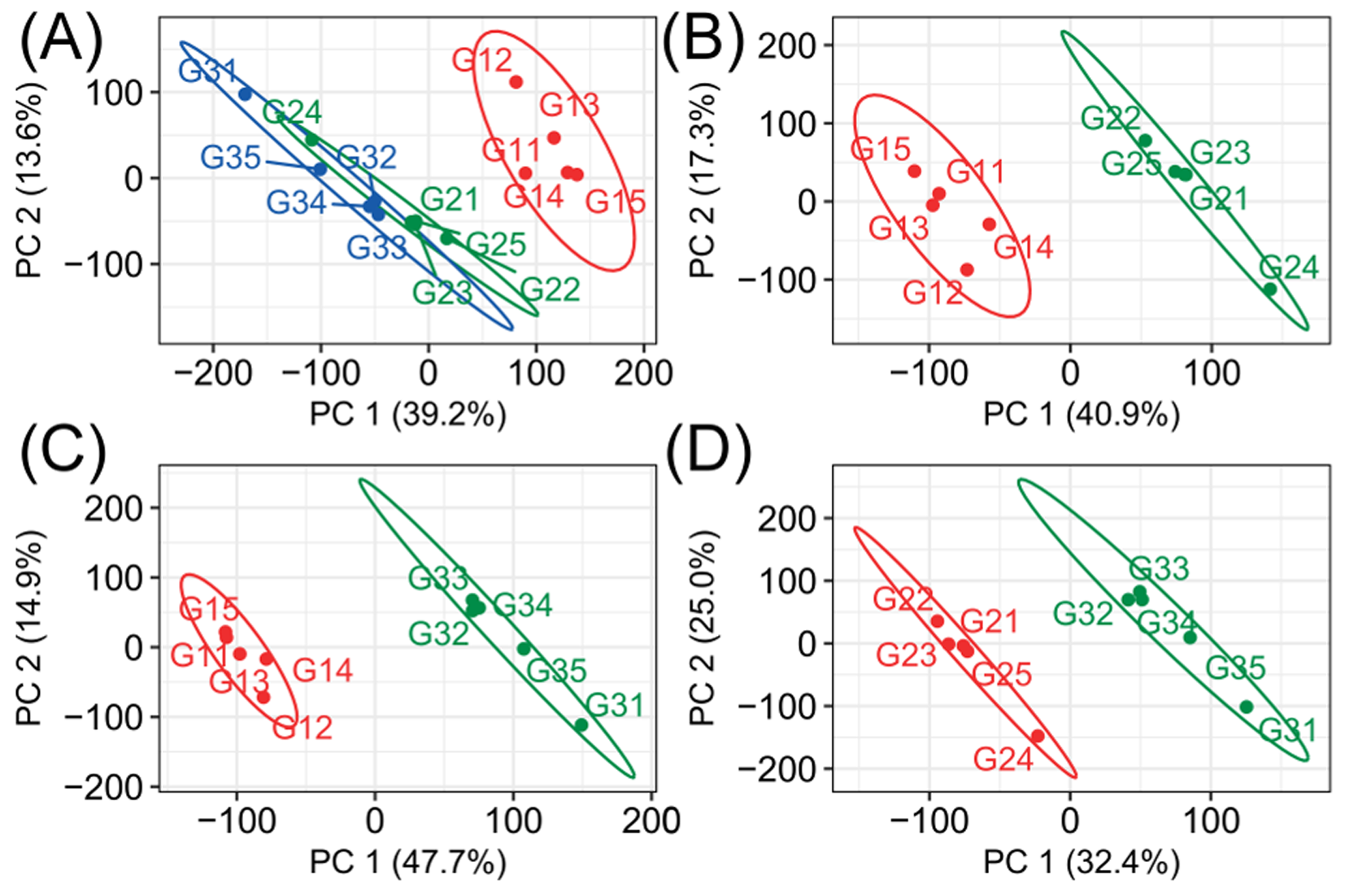

Supplement: S1 Fig — (A): Score plots based on the first two components (PC1 vs PC2) derived from the PCA result. (B), (C), and (D): Score plots based on the first two latent components derived from the corresponding PLS-DA model for the three comparisons (G2 vs G1, G3 vs G1 and G3 vs G2). The paired comparison suggested that the separation between every two groups was clear. The R2X, R2Y, Q2Y and RMSEE in PLS-DA models for groups G2 and G1 were 58.2%, 98.9%, 94.9% and 0.013, respectively. The R2X, R2Y, Q2Y and RMSEE in PLS-DA models for groups G3 and G1 were 62.6%, 99.6%, 98% and 0.039, respectively. The R2X, R2Y, Q2Y and RMSEE in PLS-DA models for groups G3 and G2 were 57.4%, 99.6%, 94.5% and 0.037, respectively. The ellipse for each group represented the Hotelling’s T2 95% confidence interval. (TIF) [file pone.0177464.s001.tif]

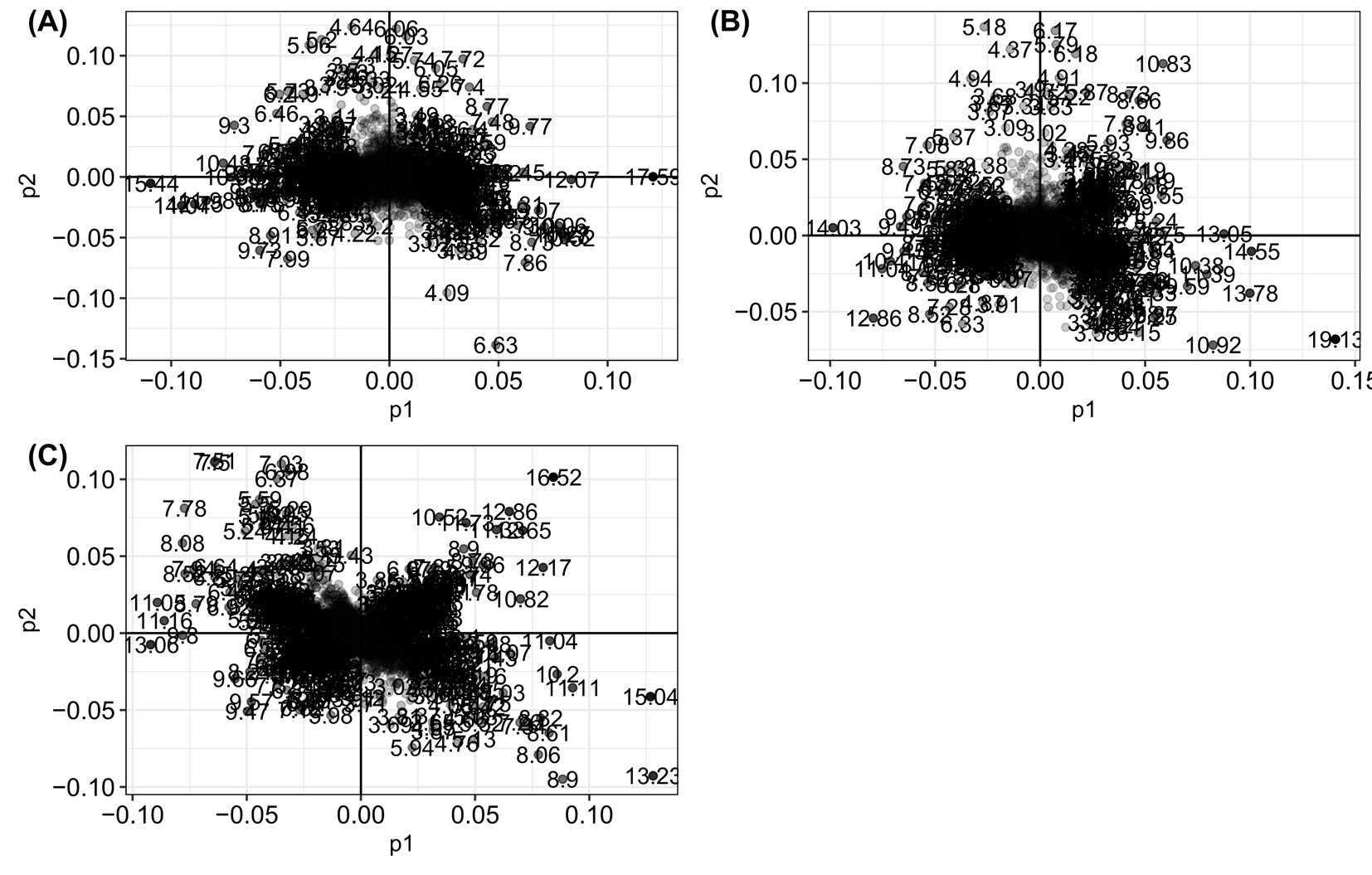

Supplement: S2 Fig — (A), (B), and (C): Loading plots based on the first two latent components derived from the corresponding PLS-DA model for the three comparisons (G2 vs G1, G3 vs G1 and G3 vs G2). The points which exhibited strong contribution to the construction of each PLS-DA model were labeled with the VIP values (VIP > 3). (TIF) [file pone.0177464.s002.tif]

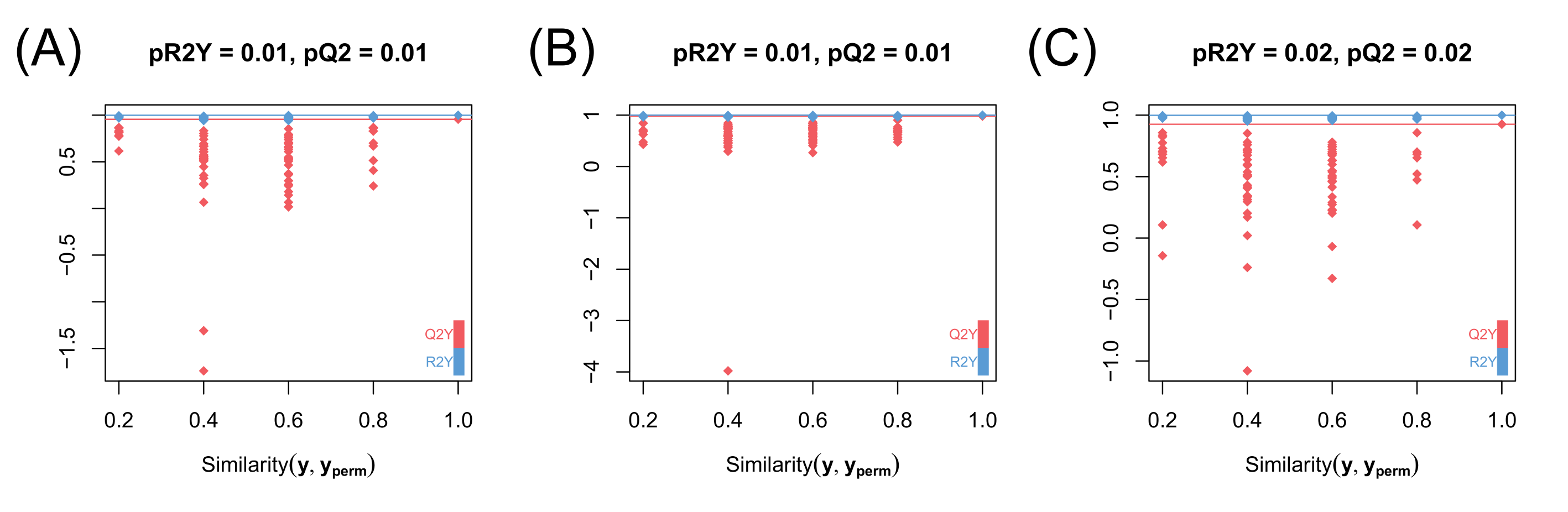

Supplement: S3 Fig — (A), (B) and (C) were presented based on the permutation tests for PLS-DA models (G2 vs G1, G3 vs G1 and G3 vs G2), respectively. The R2Y and Q2Y values in each model were proved to be significant, which suggested that each PLS-DA model was not over-fitted. (TIF) [file pone.0177464.s003.tif]
